# Manganese Toxicity in Sugarcane Plantlets Grown on Acidic Soils of Southern China

**DOI:** 10.1371/journal.pone.0148956

**Published:** 2016-03-29

**Authors:** Yu Lan Huang, Shu Yang, Guang Xia Long, Zun Kang Zhao, Xiao Feng Li, Ming Hua Gu

**Affiliations:** State Key Laboratory for Conservation and Utilization of Subtropical Agro-bioresources, College of Agriculture, Guangxi University, Nanning, China; United States Department of Agriculture, UNITED STATES

## Abstract

Ratoon sugarcane plantlets in southern China have suffered a serious chlorosis problem in recent years. To reveal the causes of chlorosis, plant nutrition in chlorotic sugarcane plantlets and the role of manganese (Mn) in this condition were investigated. The study results showed that the pH of soils growing chlorotic plantlets ranged from 3.74 to 4.84. The symptoms of chlorosis were similar to those of iron (Fe) deficiency while the chlorotic and non-chlorotic plantlets contained similar amount of Fe. Chlorotic plantlets had 6.4-times more Mn in their leaf tissues compared to the control plants. There was a significantly positive correlation between Mn concentration in the leaves and the exchangeable Mn concentration in the soils. Moreover, leaf Mn concentration was related to both seasonal changes in leaf chlorophyll concentration and to the occurrence of chlorosis. Basal stalks of mature sugarcanes contained up to 564.36 mg·kg^-1^ DW Mn. Excess Mn in the parent stalks resulted in a depress of chlorophyll concentration in the leaves of sugarcanes as indicated by lower chlorophyll concentration in the leaves of plantlets emerged from basal stalks. Ratoon sugarcane plantlets were susceptible to chlorosis due to high Mn accumulation in their leaves (456.90–1626.95 mg·kg^-1^ DW), while in planted canes chlorosis did not occur because of low Mn accumulation (94.64–313.41mg·kg^-1^ DW). On the other hand, active Fe content in chlorotic plantlets (3.39 mg kg^-1^ FW) was only equivalent to 28.2% of the concentration found in the control. These results indicate that chlorosis in ratoon sugarcane plantlets results from excessive Mn accumulated in parent stalks of planted cane sugarcanes grown on excessive Mn acidic soils, while active Fe deficiency in plantlets may play a secondary role in the chlorosis.

## Introduction

Manganese (Mn) is an essential element for plant growth but in excess, especially in acidic soils, it can become phytotoxic. The bioavailability of Mn increases as soil pH decreases below 5.5 [1, 2]. In such conditions, Mn is easily taken up by plant roots and Mn toxicity may become one of main factors limiting crop production [3]. Excessive Mn can prevent the uptake and translocation of other essential elements such as Ca, Mg, Fe, and P; inhibit chlorophyll biosynthesis; cause a decline in the photosynthesis rate; and cause an increase in the accumulation of oxidized Mn and oxidized phenolic compounds in the apoplast of leaves [4–8]. The symptoms of Mn toxicity vary widely among plant species; necrotic brown spotting on leaves, petioles and stem is a common symptom [9–11]. Another common symptom, ‘crinkle-leaf’, which occurs in the youngest leaf, stem and petiole tissues, is associated with chlorosis and browning of these tissues [10, 12].

Sugarcane, the most important sugar-yielding crop in the world, is mainly grown in tropical and subtropical regions, including Brazil, India, and China. In these countries, acidic soils cover most of the land and soil acidification has accelerated in agro-ecosystems during recent decades [13]. In China, sugarcane is mainly planted on subtropical acidic soils. In recent years, sugarcane plantlets grown in acidic soils in southern China have suffered from severe chlorosis. The planting area of sugarcane in regions exhibiting sugarcane chlorosis accounts for approximately 40% of the total sugarcane acreage in China. The chlorosis has resulted in a reduction of cane production ranging between 23% and 40%. These soils belong to the red soils derived from quaternary clay or sandy shale. The symptoms of chlorosis were similar to those of Fe deficiency. In the present paper, we report the development of the disorder in sugarcane plantlets and its relationship with excessive Mn in plants grown in the acidic soils of southern China.

## Materials and Methods

### Investigation of the status of chlorosis in sugarcane, plant nutrients and soil chemicals

From 2008 to 2014, surveys on the status of chlorosis in sugarcane plantlets were conducted in the cities of Chongzuo, Nanning, Laibin, and Guigang, located in Guangxi Province, China. These cities are the major regions of sugarcane production in China and large acreage of sugarcanes in these regions occur chlorosis in spring. Symptoms of chlorosis were photographed (D7000 camera, Nikon Co., Thailand).

To investigate the involvement of plant nutrients in the chlorosis of sugarcane plantlets grown in various areas ranging from 22°30′36″ to 22°43′6″ N latitude and 107°31′24″ to 109°24′12″ E longitude, plant samples were collected from chlorotic and non-chlorotic (control) plantlets during March and April 2014. Soil samples were also collected from the fields where plant samples were collected. Fifty-eight widely situated fields, 29 where ratoonsugarcane chlorosis occurs consistently year-after-year and another 29 adjacent to chlorotic regions where the problem had never occurred in the past, were selected for sample collection in the four cities listed above. Other selection criteria were that the fields had the crop similar in age and that the growers had not applied any foliar spray to the crop.

Plant samples were collected randomly from the first expanded leaf and 20 leaves were included in per sample (and also in samples described below). Chlorophyll and active Fe concentrations were measured after the leaves were cleaned andcut into 0.5 mm segments with stainless steel scissors. Chlorophyll was extracted from leaf segments with 80% acetone for 24 h in the dark, and determined spectrophotometrically (Shimadzu UV-1800, Kyoto, Japan) according to the methods of Arnon [14]. Active Fe in the leaves was determined by AAS after leaf-segments were extracted with 1 mol L^-1^ HCl according to the methods of Mehrotra and Gupta [15]. Concentrations of total Mn, N, S, Fe, Mg, Cu, and Zn were also analyzed after the leaves were cleaned, dried and crushed. Plant samples were microwave-digested in pressurized perfluoroalkoxy alkane vessels containing a mixed acid solution (3:1 HNO_3_:HCl). Concentrations of Fe, Mn, Zn and Mg in the digested solution were measured by an atomic absorption spectrometer (AAS) (700 P, Thermo Co., Germany). The concentration of S in the digested solution was measured by ionic chromatography (ICS-5000, Dionex Co., CA, USA). For determination of N, the plant samples were digested with H_2_SO_4_ using H_2_O_2_ as a catalyst [16]. Nitrogen concentrations in the digested solutions were determined with a continuous flow analytical system (AA3, Bran-Luebbe Inc., Germany).

Soil samples were collected from 0–20 cm of the topsoil layer. Soil pH values were determined after soil samples were air-dried, sieved (2 mm) and suspended in purified water with a soil to water ratio of 1:2.5 [17]. Exchangeable Mn in these samples was measured by AAS after the soils were extracted with 1 mol L^-1^ ammonium acetate (pH 7.0) for 30 min [18].

### Field collection of leaves from chlorotic and non-chlorotic plantlets of ratoon sugarcanes for Mn and chlorophyll analyses

Leaf sampleswere collected, respectively, from chlorotic plantlets and adjacent, non-chlorotic plantlets of ratoon sugarcane grown in chlorosis affected fields on three farms, where the chlorosis is the most serious in above cities, in Wangzhuang (WZ) ((22°36′21″ N, 107°51′15″ E), Balian (BL) ((22°45′13″ N, 107°55′4″ E), and Changpin (CP) (22°44′51″ N, 107°54′60″ E), rural areas of Chongzuo.

### Field collection of leaves from plantlets of planted cane and ratoon sugarcanes for Mn and chlorophyll analyses

Samples of the first fully expanded leaf, leaves at the upper position (top leaves) and leaves at the lower position (other leaves) were collected respectively from the plantlets of planted cane sugarcanes and ratoon sugarcanes grown in a field where ratoon sugarcane chlorosis occurs consistently year-after-year. The field was located in Chongzuo (22°35′36″ N, 107°51′19″ E).

### Field collection of leaves from plantlets of planted cane and ratoon sugarcanes to monitor seasonal variation in Mn and chlorophyll concentrations

Samples of the first fully expanded leaf were collected from planted cane sugarcanes and ratoon sugarcanes, respectively, on July 7, September 25, November 23 of 2012, and March 29, May 6, June 19, July 23, and November 25 of 2013. Sugarcanes were planted in 2012 in a field where ratoon sugarcane chlorosis occurs consistently year-after-year in Chongzuo (22°35′36″ N, 107°51′19″ E). Cane stalks of the planted sugarcane were harvested in January 2013. In February and March 2013, 2–3 months later, plantlets had emerged from underground basal stalks of the above planted and harvested sugarcanes.

### Investigation of Mn concentration in basal cane stalks

To investigate differences in the Mn concentration between the basal stalks and stalk segments at other positions for mature planted cane sugarcane, we collected 20 sugarcanes grown in a field of Chongzuo (22°35′36″ N, 107°51′19″ E), where ratoon sugarcane chlorosis occurs consistently, on November 3, 2014. The canes were cut into segments containing two stem internodes each. From top to bottom, the segments were named top, first, second, third, fourth, fifth, and Base.

### Culture experiment

To investigate the impact of Mn in parent cane on the leaf chlorophyll concentration of plantlets, we collected sugarcane stalks of mature planted cane sugarcane from a Chongzuo sampling site (22°35′36″ N, 107°51′19″ E) where ratoon sugarcane chlorosis occurs consistently year-after-year. According to the number of internodes per stalk, each stalk was cut into three equal segments (upper, middle, and lower) and cultured in plastic containers (five segments per container). The bottom of the container was covered with towels saturated with deionized water. Cane segments were placed on towels and were covered by moist filter papers and sponges to maintain moisture levels. Deionized water was sprayed on filter papers and sponges every day to maintain consistent moisture levels. The plants were cultured in an environment-controlled growth room with a 14-h/28°C day and 10-h/20°C night cycle and a light intensity of 45 μmol m^-2^ s^-1^. After 30 days, all leaves of plantlets were collected to determine their chlorophyll concentrations.

### Ethics statement

All experiments carried out in this study comply with current laws of China. Soil and plant samples were collected with oral permission from farmers or landowners to avoid interference with normal agriculture activities. No other permits were required according to Chinese law for this commonwealth project which provides potential benefit to farmers, citizens, and the local government. The field studies did not involve endangered or protected species.

## Results

### Chlorosis in sugarcane plantlets

**Fig 1:**
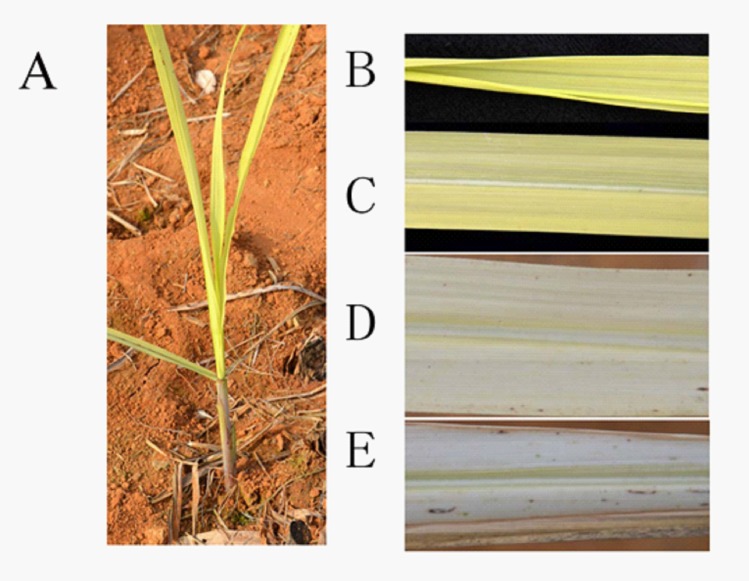
Chlorotic sugarcane plantlets (A), spreading leaf (B), and spread leaf (C, D, E) showing yellowing on the whole spreading leaf (B) and the first spread leaf (C), yellow-white leaf (D), and margin necrotized and withered leaf (E).

Visual symptoms of chlorosis in sugarcane plantlets resemble those of Fe deficiency. The incidence of chlorosis differed distinctly between planted cane sugarcane plantlets and ratoon plantlets. Ratoon plantlets clearly showed chlorosis symptoms. On the contrary, planted sugarcane did not exhibit chlorosis symptoms. A pale yellow chlorosis first appeared in the emerging leaves at the ratoon plantlet stage (Fig 1A). Yellow chlorosis subsequently developed in spreading leaves (Fig 1B). As the chlorosis increased in severity, the lower leaves became yellowish-white and their margins necrotized and withered (Fig 1C, 1D and 1E). Progressively, the whole plant became yellowish-white and the chlorotic leaves finally dried. The yellow chlorosis and subsequent leaf drying progressed upwards until the terminal leaf became dry and the plants died prematurely. Ultimately, the mortality rate of chlorotic ratoon plantlets in the fields reached approximately 50%.

The plantlet stage was the sole stage of ratoon sugarcane showing symptoms of chlorosis. At the late plantlet stage, the overall yellowing leaves of the surviving plantlets gradually demonstrated only intervenal chlorosis, and ultimately the symptoms of chlorosis disappeared completely. The veins of the lower leaves greened first, followed by intervenal areas of the leaves. Progressively, the upper leaves greened. Ultimately, the whole plantlets greened and no visible chlorosis was observed after the early tillering stage.

### Chlorophyll in leaves

**Fig 2:**
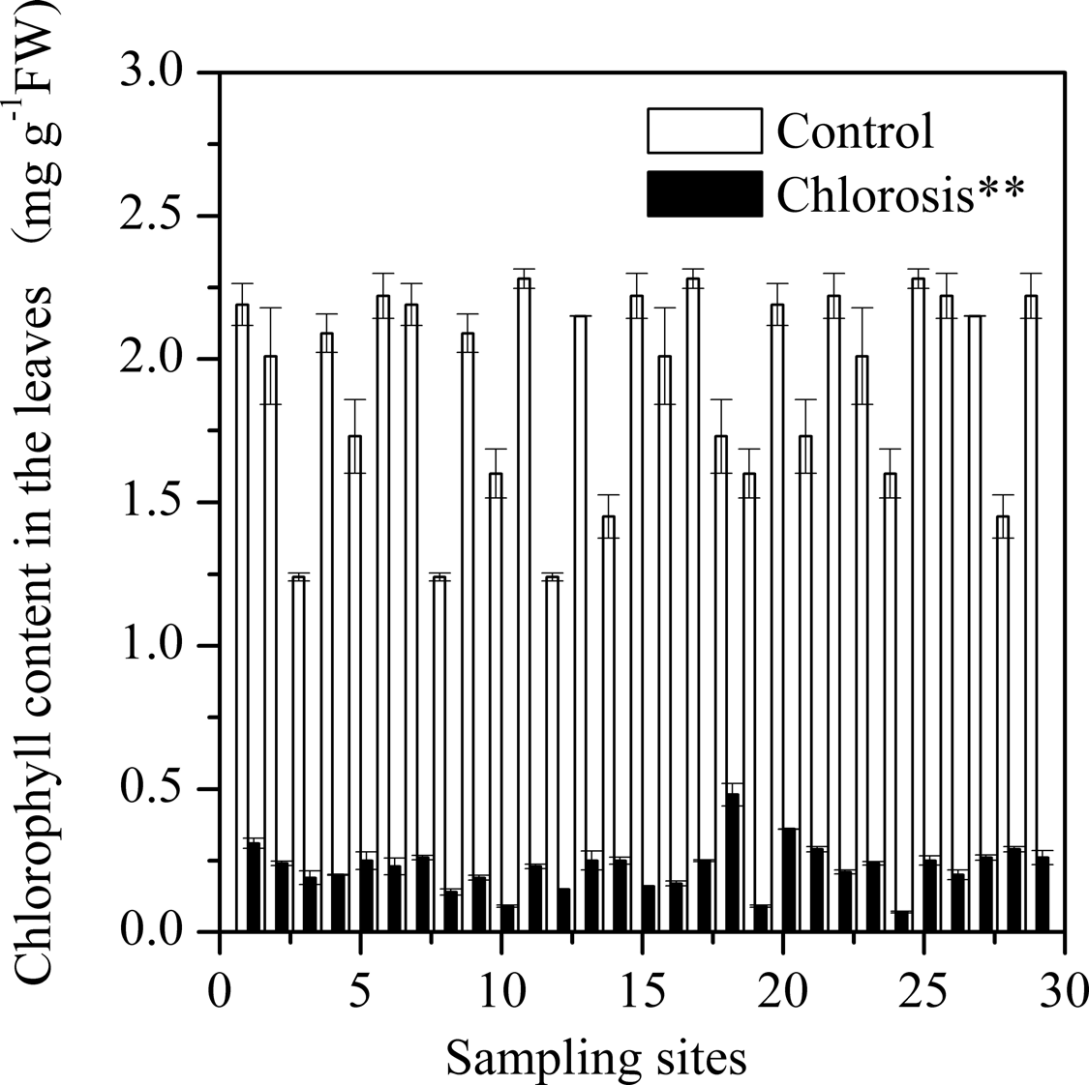
Chlorophyll concentrations in sugarcane leaves of chlorotic plantlets grown in chlorosis affected fields and non-chlorotic plantlets (control) grown in chlorosis unaffected fields. Each value is the mean ± SEM (n = 3 replicates). Pairwise Student’s *t* test was used to compare value to the control. **, Significant at P = 0.01.

The leaves of chlorotic plantlets contained a low concentration of chlorophyll. The chlorophyll concentration in the leaves of chlorotic plantlets collected from different sites ranged from 0.07 ± 0.00 mg g^-1^ FW (fresh weight) to 0.48 ± 0.04 mg g^-1^ FW, all significantly lower than those in the control plants from the same geographicsites (Fig 2). The average chlorophyll concentration of chlorotic plantlets (0.23 ± 0.02 mg g^-1^FW) was equivalent to just 11.8% of that found in control plants (1.92 ± 0.07 mg g^-1^FW).

### Development of chlorosis and accumulation of Mn in the plantlets

**Fig 3:**
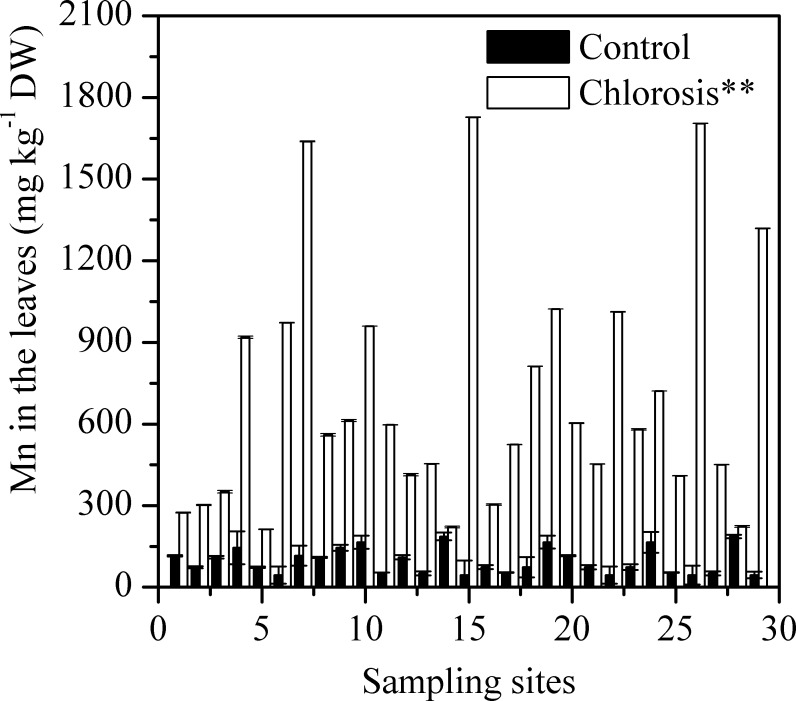
Mn concentration in the leaves of chlorotic and non-chlorotic plantlets. Each value is the mean ± SEM (n = 3 replicates). Pairwise Student’s *t* test was used to compare value to the control. **, Significant at P = 0.01.

**Fig 4:**
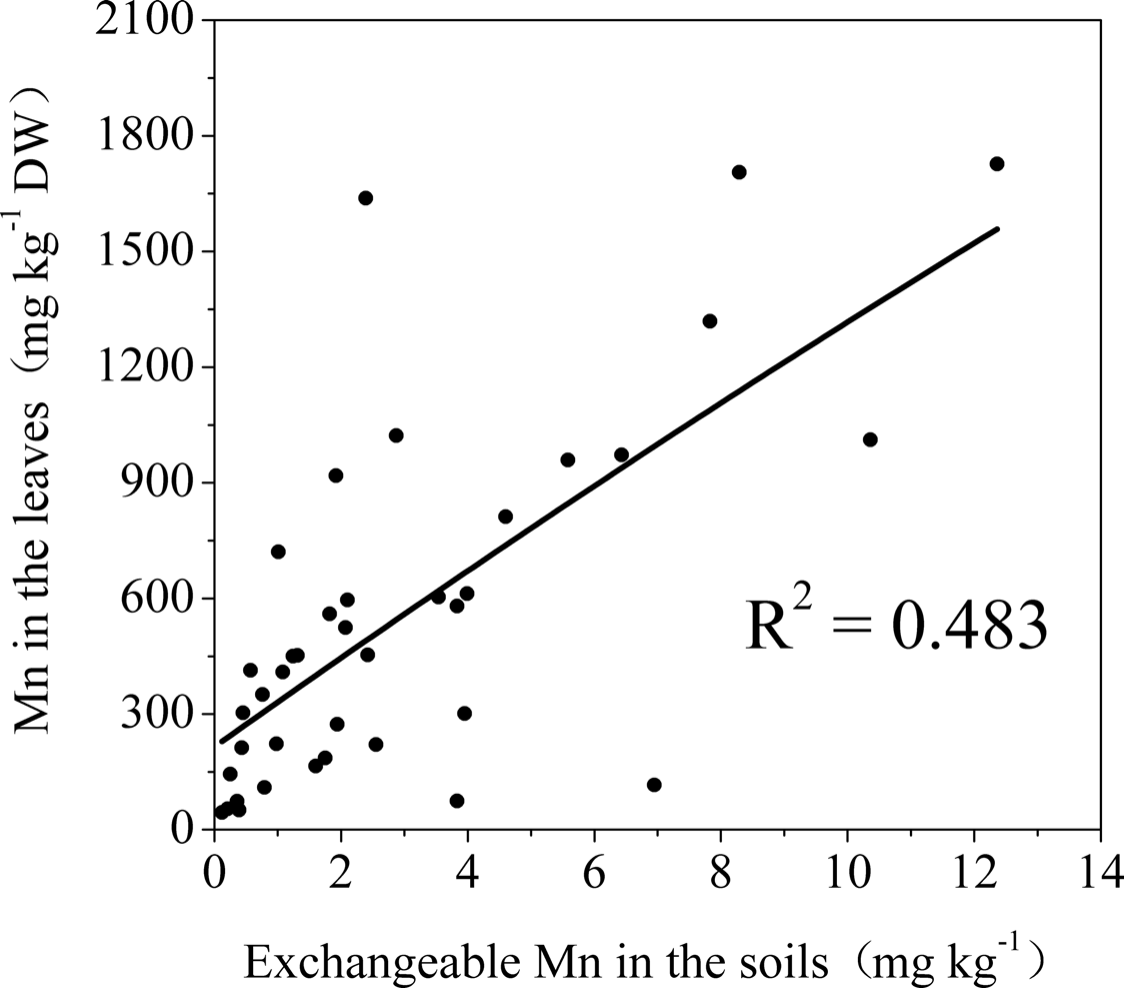
The relationship between Mn concentration in the leaves of sugarcane plantlets and exchangeable Mn in the corresponding soils. ** indicates statistical significance (p<0.01) according to correlation pertinent statistic.

Chlorotic plantlets all contained high concentrations of Mn (Fig 3). The Mn concentration in the leaves of control plants was between 44.53 ± 0.99 and 186.31 ± 3.34 mg kg^-1^, and the mean was 95.29 ± 8.79 mg kg^-1^ (Table 1). The Mn concentrations of chlorotic plantlets from different sampling sites varied between 212.77 ± 14.22 and 1727.16 ± 53.14 mg kg^-1^, and the mean was 701.67 ± 82.22 mg kg^-1^. Thus, the mean Mn concentration in chlorotic plantlets was 6.4 times greater than that in the control plantlets. There was a significant positive correlation between Mn concentration in the plantlets and the concentration of exchangeable Mn in the soil (Fig 4).

**Table 1:** Concentrations of N, Mg, S, Zn, Fe and Mn in the leaves of chlorotic and non-chlorotic (control) sugarcane plantlets at various sites. Pairwise Student's *t*-test was used to compare values to the control.

Item	N (g·kg^-1^ DW)		Mg (g·kg^-1^ DW)		S (g·kg^-1^ DW)		Zn (mg·kg^-1^ DW)		Fe (mg·kg^-1^ DW)		Mn (mg·kg^-1^ DW)	
	Control	Chlorosis	Control	Chlorosis	Control	Chlorosis	Control	Chlorosis	Control	Chlorosis	Control	Chlorosis
Range	14.27–21.79	14.17–20.93	1.02–1.56	0.95–1.67	1.14–4.07	0.87–4.73	9.18–25.49	15.91–40.58	130.07–218.31	87.77–248.62	445.53–186.31	212.77–1727.16
Mean	18.23	17.15*	1.22	1.33*	3.03	2.80	16.03	21.73**	173.21	158.68	95.25	701.67**

*Significant at p = 0.05 (n = 29).

**significant at p = 0.01 (n = 29).

**Fig 5:**
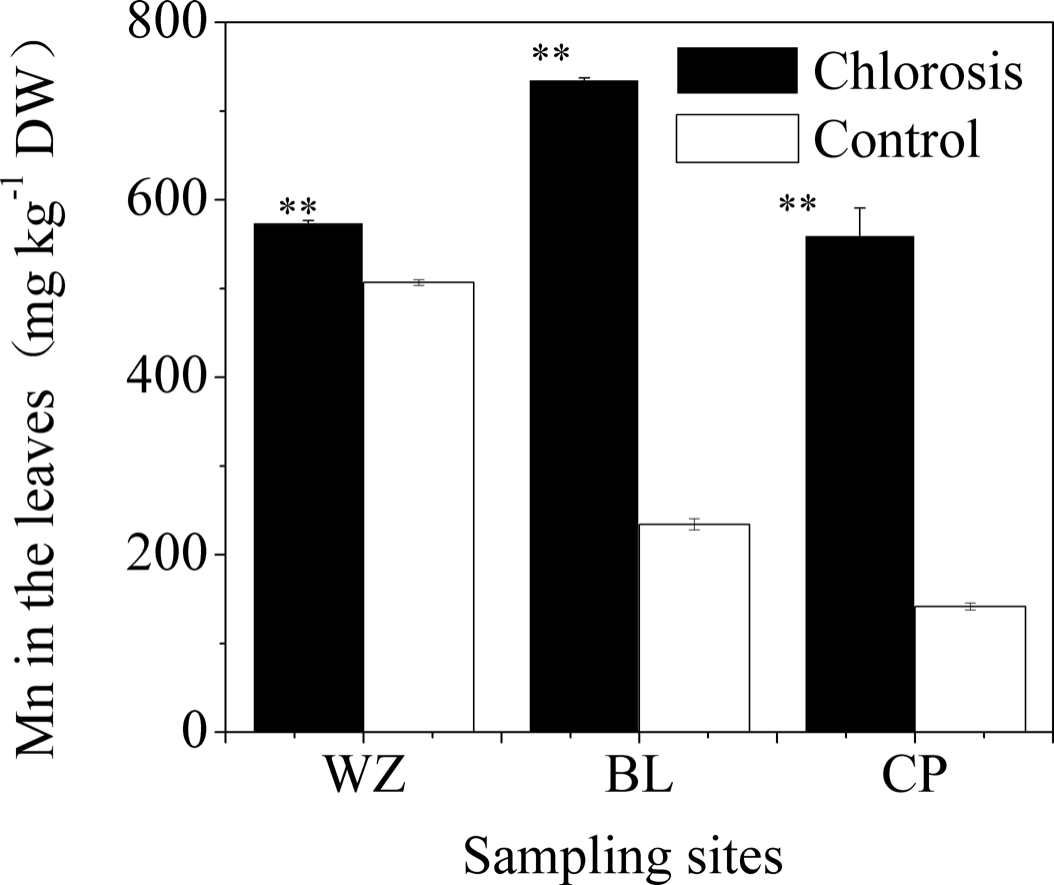
Mn concentration in the leaves of chlorotic plantlets and neighboring plantlets (Control) without chlorosis symptoms. Each value is the mean ± SEM (n = 3 replicates). Pairwise Student’s *t* test was used to compare value to the control. **, Significant at P = 0.01.

In parts of survey areas, the degree of chlorosis among plantlets was not uniform, and there were even significant differences between neighboring plants (S1 Fig). The plantlets with high Mn concentration collected from the WZ, BL, and CP sampling fields exhibited chlorosis, but plantlets which were close to chlorotic plantlets and had low concentrations of Mn did not exhibit chlorosis (S1 Fig and Fig 5).

The leaves of ratoon sugarcane contained higher concentrations of Mn but lower concentrations of chlorophyll than planted cane sugarcane (Table 2); as a result, the plantlets exhibited visual symptoms of chlorosis (S2 Fig). In contrast, the leaves of planted cane sugarcanes planted in a field adjacent to ratoon plantlets contained lower concentrations of Mn but higher concentrations of chlorophyll; thereby, the planted cane plantlets did not exhibit obvious chlorosissymptoms. These results indicate that the differences in susceptibility to chlorosis between planted cane plantlets and ratoon plantlets were determined by the Mn concentration in the plantlets.

**Table 2:** Comparison of the concentrations of chlorophyll and Mn in leaves between ratoon sugarcanes and planted cane sugarcanes grown in achlorotic field in Chongzuo, Guangxi, China. Pairwise Student's *t*-test was used to compare values to planted cane sugarcane.

Leaf position	Chlorophyll (mg g^-1^ FW)		Mn (mg·kg^-1^ DW)	
	Planted cane	Ratoon	Planted cane	Ratoon
Top leaves	1.48±0.04	0.26±0.01*	94.64±1.01	456.90±5.86*
The first spread leaf	2.13±0.01	0.29±0.01*	191.21±7.51	1023.20±44.22*
Other leaves	2.89±0.12	0.6±0.07*	313.41±3.60	1626.95±6.52*

*Significant at p = 0.05 (n = 3).

**Fig 6:**
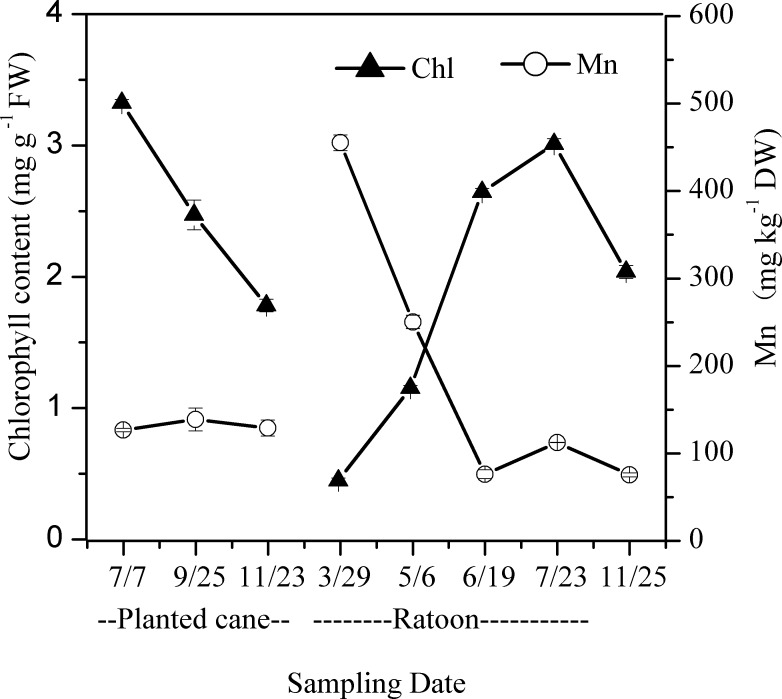
Time course of chlorophyll and Mn concentration alternation in the leaves of planted cane and ratoon sugarcanes, which were emerged on the basal stalks of the planted cane sugarcane. The leaves were collected on Jul 7, Sep 25, and Nov 23 of 2012 and Mar 29, May 6, Jun 19, Jul 23, and Nov 25 of 2013, respectively. Each value is the mean ± SEM (n = 3 replicates).

The Mn and chlorophyll concentrations in the leaves of ratoon sugarcanes varied with sugarcane growth stages and seasons (Fig 6). From the elongation stage to the maturity stage (July to November), the Mn concentrations in the leaves of planted cane sugarcanes and ratoon sugarcanes were low, while the chlorophyll concentrations in the leaves were high. In the early plantlet stage (March, 2013), the Mn concentration in the leaves of ratoon sugarcanes exhibited a substantial increase compared with their parent plants (July to November, 2012), which were planted cane sugarcanes planted in the previous year. In the early plantlet stage, the chlorophyll concentration in the leaves exhibited a substantial decrease relative to their parent plants and the plantlets exhibited severe symptoms of chlorosis. After entering the late plantlet stage and the early tillering stage (May), the chlorophyll concentration in the leaves of surviving ratoon plantlets increased rapidly. During the greening process of chlorotic leaves, the changes in leaf Mn and chlorophyll concentrations showed opposite trends.

**Fig 7:**
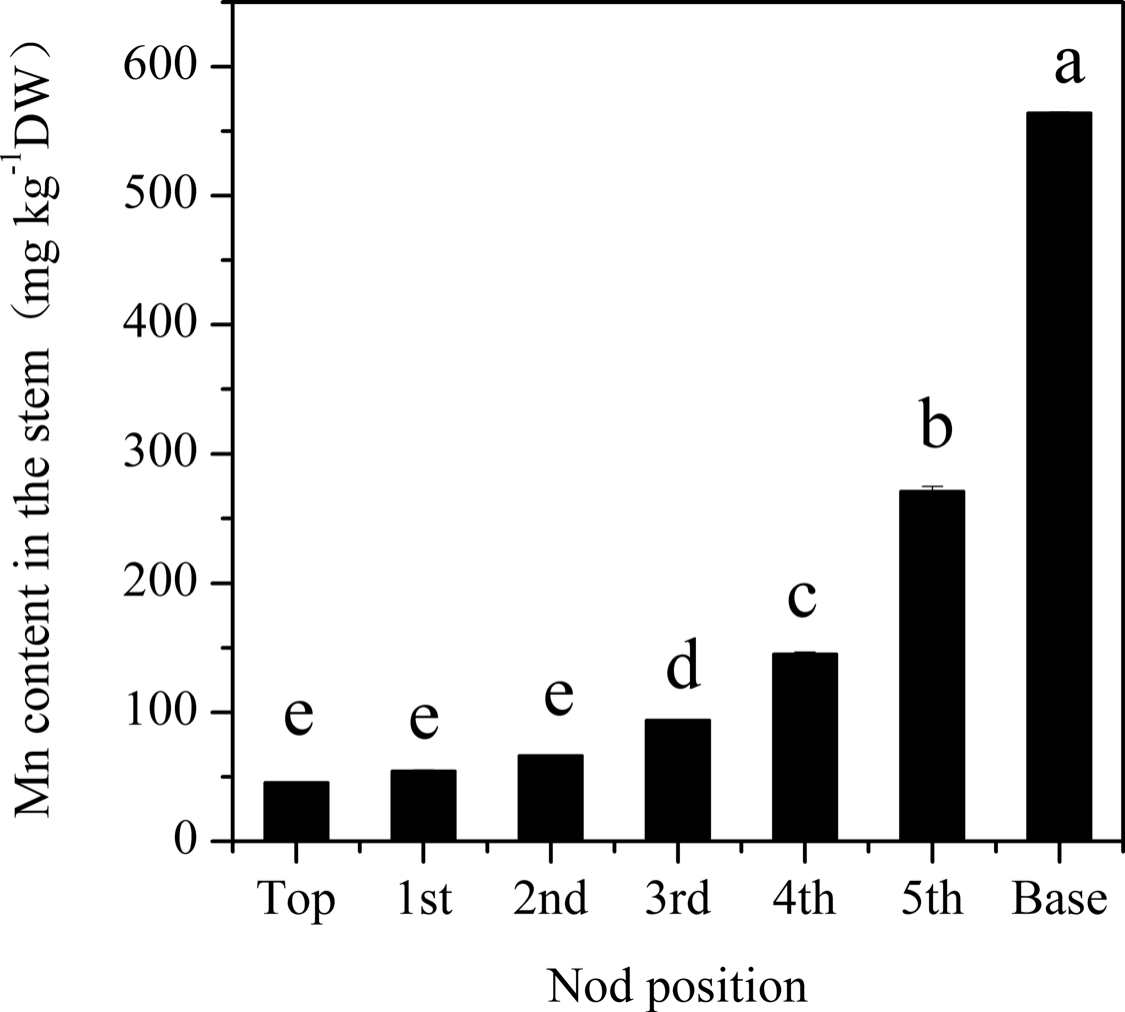
Mn concentration in the top first, second, third, fourth, fifth and base segments of canes at the mature stage of sugarcanes grown on strongly acidic soil with a high concentration of exchangeable Mn. Each value is the mean ± SEM (n = 3 replicates). Different letters on the columns indicate that the values are significantly different at the 0.01 level, according to Tukeyʼs Multiple Range Test.

**Fig 8:**
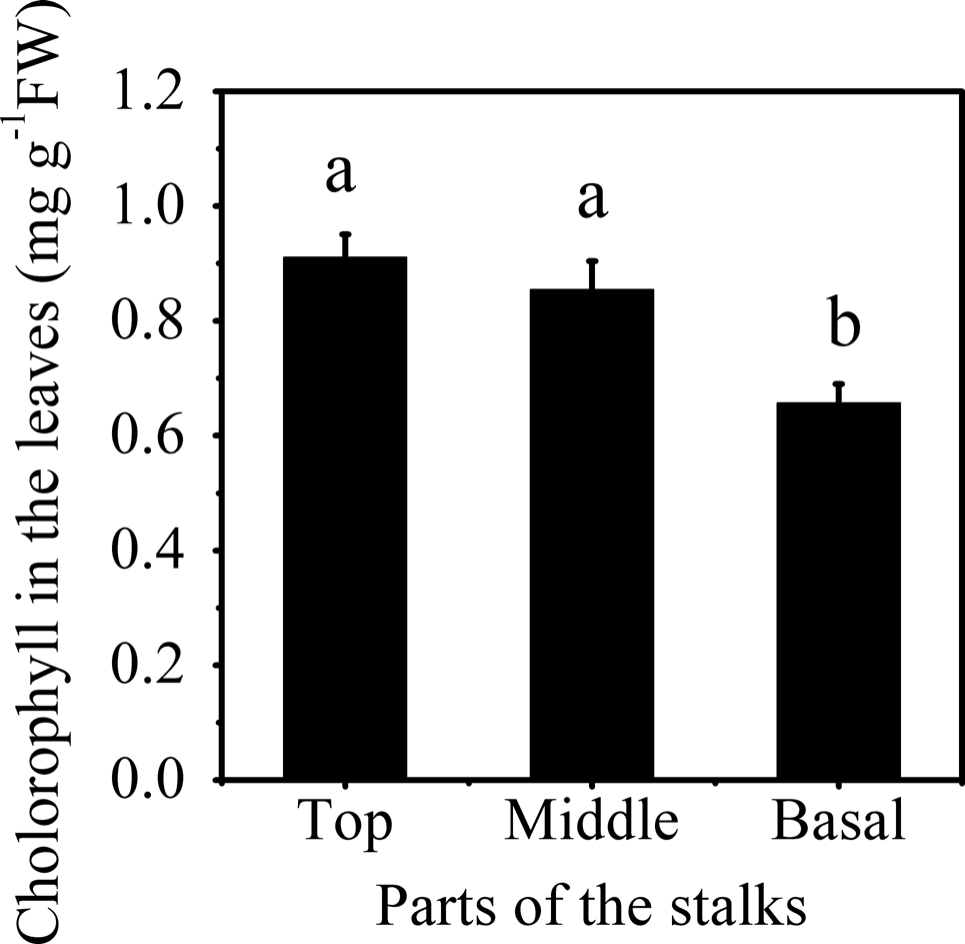
Chlorophyll concentration in the leaves of sugarcane plantlets emerged from the top, middle, and basal parts of parent stalks. Different letters on the columns indicate that the values are significantly different at the 0.05 level, according to Tukey’s Multiple Range Test.

The Mn concentration in different stalks of planted cane sugarcanes at the mature stage significantly increased with the decline in their internode position (Fig 7). The Mn concentration was highest in basal stalks, reaching 564.36 ± 0.68 mg kg^-1^DW (dried weight), which was 11.3 times greater than that in top stalks. After being cultured in deionized water containing no Mn, plantlets emerged from the basal stalks of planted cane sugarcanes had a significantly lower amount of chlorophyll in their leaves (Fig 8), equivalent to 76.3% and 72.1% respectively, of the amounts found in the plantlets emerged from the middle and top stalks. These results indicated the role of parent stalk Mn in the depression of chlorophyll concentration in ratoon plantlets.

### Fe in plantlets

**Fig 9:**
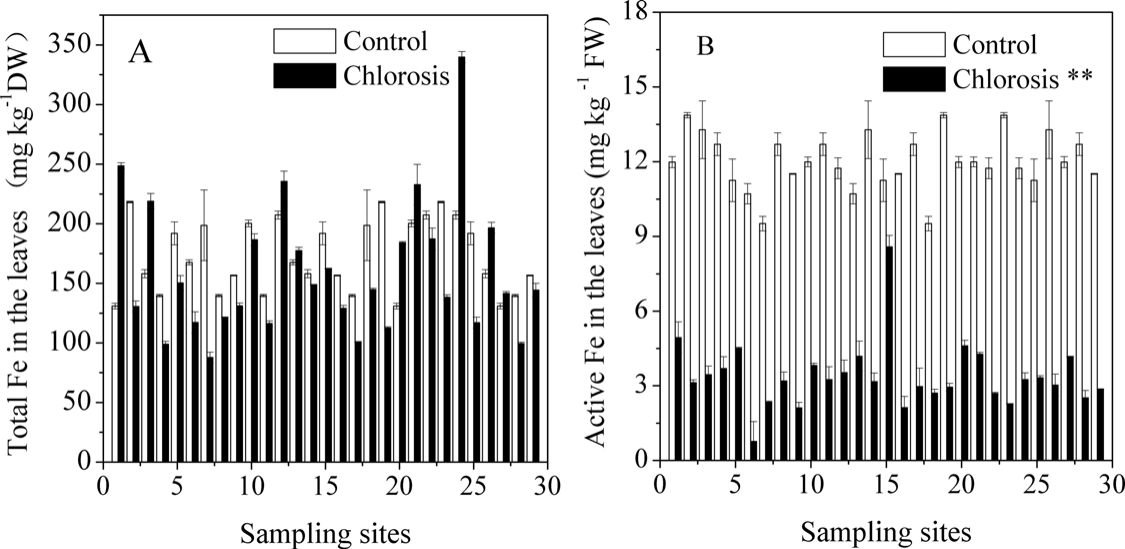
Total Fe (A) and active Fe (B) concentration in leaves of chlorotic and control plantlets grown at 29 sites in various areas. Each value is the mean ± SEM (n = 3 replicates). Pairwise Student’s *t* test was used to compare value to the control. **, Significant at P = 0.01.

The total Fe in chlorotic plantlets was within the normal concentration range. The total Fe concentrations in the leaves of chlorotic plantlets from different sampling sites and in the leaves of control plants ranged between 87.77 ± 4.44–248.62 ± 2.61 mg kg^-1^ DW and 131.07 ± 2.39–218.31 ± 0.69 mg kg^-1^ DW, respectively (Table 1). Although the symptoms of chlorosis in sugarcane plantlets were similar to those of Fe deficiency, the difference in Fe concentration between chlorotic plantlets and the control plants was not significant (Fig 9A). Among the 29 chlorotic samples, the Fe concentrations of nine were even higher than in their respective controls. The plantlets with the highest Fe concentration (248.62 ± 2.61mg kg^-1^ DW) exhibited significant chlorosis.

HCl extracted Fe reflects the concentration of active Fe in plantlets. Active Fe concentration in chlorotic plantlets was significantly lower than in control plants (p < 0.01) (Fig 9B). The active Fe concentrations of chlorotic plantlets collected from different sampling sites varied between 0.77–8.58 mg kg^-1^ FW, which was 7.2%–76.2% of that found in the control plants, respectively. The average active Fe concentration in chlorotic plantlets was 3.39 mg kg^-1^ FW, only equivalent to 28.2% of the concentration found in the controls (12.03 mg kg^-1^ FW).

### S, N, Mg and Zn in the plantlets

The concentration of S in chlorotic plantlets was comparable to that in the control plants (Table 1). Although the concentrations of Mg and Zn were significantly different between chlorotic plantlets and control plants, the concentrations were higher in chlorotic plantlets than in controls. Although there was a significant difference in leaf N concentration between chlorotic plantlets and the control plantlets, the average N concentration in chlorotic plantlet leaves was 17.155 g.kg^-1^, equivalent to 94.1% of the concentration in control plantlets.

### Soil acidity

**Fig 10:**
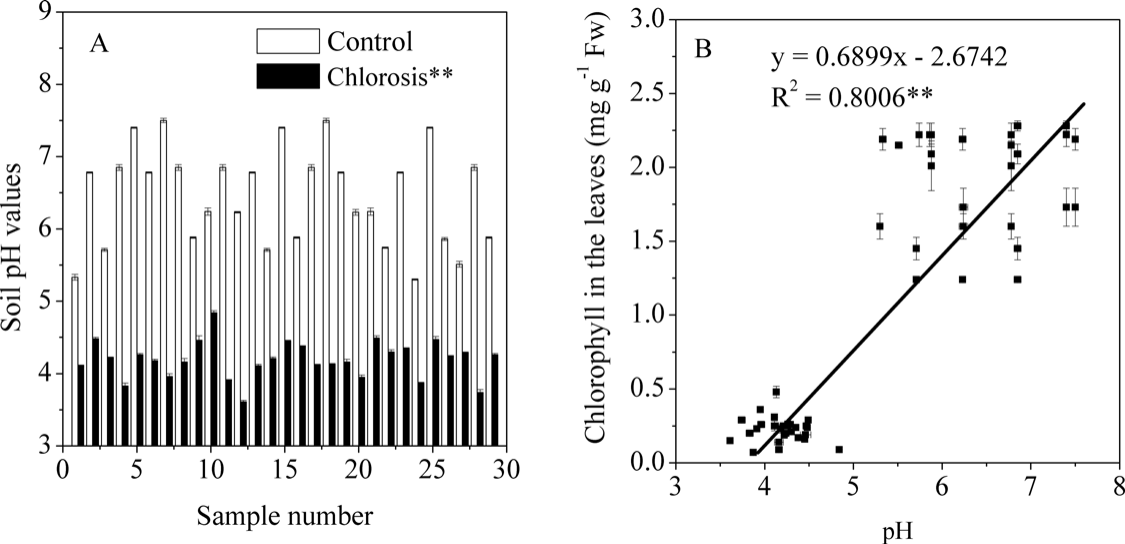
The pH values of soils collected during March and April 2014 from various sites where chlorotic or non-chlorotic sugarcane plantlets were grown (A) and the relationship between chlorophyll concentration in the leaves of sugarcane plantlets and the pH value of the corresponding soil (B). Each value is the mean ± SEM (n = 3 replicates). Pairwise Student’s *t* test was used to compare value to the control. **, Significant at P = 0.01.

The soil from which chlorotic samples were obtained ranged from pH 3.61 to 4.84, and most of the soils were strongly acidic (Fig 10A). In contrast, the pH values of the control soils ranged between5.30 and 7.50. Plantlets grown in control soils with pH >5.30 did not exhibit symptoms of chlorosis.Correlation analysis showed that the soil pH value had a significant positive correlation with the concentration of chlorophyll in the leaves of sugarcane plantlets (Fig 10B).

## Discussion

### Chlorosis of ratoon sugarcane plantlets grown in acidic soils was resulted from excessive Mn

Mn toxicity, as an important constraint to crop production in tropical and subtropical soils, has been well documented in plant species including rice, soybean and cowpea. However, little is known about Mn toxicity in sugarcane, despite the significance of sugarcane production in supporting global sugar supply. In the present study, we demonstrated that the chlorosis of sugarcane plantlets grown on acidic soils in southern China was caused by excessive Mn accumulation in the plants.

Mn concentration in plants is the most important and reliable index for the diagnosis of Mn phytotoxicity. Mn toxicity may occur when the Mn concentration in the above ground parts of plants reaches 150 mg kg^-1^ [4]. In the present study, we found that the Mn concentration in the leaves of chlorotic plantlets reached to 701.67 ± 82.22 mg kg^-1^ (Table 1). The Mn concentration in chlorotic plantlets was significantly higher than in non-chlorotic plantlets collected from chlorosis affected (Figs 5 and 6) and unaffected fields (Table 1 and Fig 3). The plantlets of ratoon sugarcane contained higher amount of Mn (Table 2) and exhibited obvious chlorosis. In contrast, planted cane contained less amount of Mn and sequentially did not exhibit the symptoms of chlorosis. Moreover, excessive Mn in parent stalks resulted in a depression of chlorophyll concentration in ratoon plantlets (Figs 7 and 8). Excessive Mn-induced depressions of chlorophyll concentration and chlorosis were also found in pineapple [19]. These results suggest that chlorosis in ratoon sugarcane plantlets is excessive Mn-induced phytotoxicity.

Underground basal stalks of planted cane sugarcane are parent stalks of ratoon sugarcane, while top stalks of sugarcane are parent stalks of planted cane sugarcane. Basal stalk had up to 564.36 mg·kg^-1^ DW Mn, which was 11.3-times greater than Mn in top stalks. Chlorotic plantlets of ratoon sugarcane have no living roots to uptake Mn from soils (S3 Fig). Thus, higher concentration of Mn in parent stalks should be an important reasons leading higher concentration of Mn in plantlets and more susceptibility to chlorosis in ratoon sugarcane compared with plant cane sugarcane.

N, S, Mg and Zn are component elements of chlorophyll or necessary elements for chlorophyll biosynthesis [20]. However, the concentrations of S, Mg and Zn in chlorotic plantlets were neither less than in non-chlorotic plantlets nor too high to be toxic (Table 1) [21]. The symptoms of chlorosis, which were first exhibited in expanding leaf, differed from those of N deficiency [22], although chlorotic plantlets contained less amount of N than non-chlorotic plants (Table 1). These results suggesting that the chlorosis did not result from deficiency or toxicity of N, S, Mg or Zn.

Mn toxicity to plants is governed by the soil Mn reserve and pH [20]. Each pH unit decrease elevates Mn concentration in the soil solution by 100-fold [2]. In the present study, we found that Mn toxicity of sugarcane plantlets occurred in acidic soils with a highest pH of 4.84 (Fig 10A). Similarly, Mn-induced chlorosis developed in pineapples grown on acidic soil of Mn mine tailing origin [19]. These results suggest that excessive Mn in acidic soils contributes to Mn toxicity in sugarcanes.

Crinkle-leaf and necrotic brown spotting on leaves are typical symptoms of Mn toxicity. Crinkle-leaf is associated with chlorosis of the youngest leaf, stem and petiole tissues [10, 12]. Intervenal chlorosis of young leaves is an initial symptom of Mn toxicity. When the toxicity becomes more pronounced, leaf vein also yellows, and overall chlorosis of the young leaves develops subsequently. However, in our study on sugarcane, necrotic brown spotting was hardly observed on leaves, except for the yellowing-white leaves (Fig 1). “Crinkle-leaf” did not develop on the field sugarcane. More interesting, overall yellowing of the emerging leaves and expanding leaves at the early plantlet stage were the initial symptoms of Mn toxicity, while intervenal chlorosis was a subsequent symptom that developed in the late plantlet stage. These results suggest that Mn toxicity symptoms in sugarcanes develop with a distinctive pattern.

### Possible mechanism of Mn-induced chlorosis in sugarcane plantlets

Peroxidation damageis an important indicator of Mn toxicity in plants [4, 7, 8, 23–25]. Mn^2+^ toxicity can induce oxidative stress through direct generation of reactive oxygen species (ROS) from Mn^2+^ in the Fenton reaction, or by direct transfer of electrons, leading to a rise in ROS level [25–29]. Mn^2+^ in chloroplasts can be oxidized by light-activated chlorophyll into Mn^3+^ [6, 29, 30]. The elevated reduction potential of Mn^3+^can lead to accumulationof O^2−^, H_2_O_2_, OH^.^, and other reactive free radicals, and thereby damage chlorophyll [31]. In 1950, Gerretsen proposed that Mn^2+^-induced chlorosis in higher plants could be caused by photo-oxidation of chlorophyll rather than by inhibition of chlorophyll synthesis [32]. More recently, Gonzáles et al. have suggested that Mn^2+^-toxicity could include a photo-oxidative component [29]. Excess Mn caused structural damage to chloroplast, thus hindering chloroplast function [5, 6, 29, 31, 33]. Excess Mn may substitute for Mg^2+^ in chlorophyll molecules or bind to ferredoxin in the thylakoidmatrix, thereby destroying chloroplast structure [5, 31]. These results suggest that chlorophyll damage caused by excess Mn may be one of the reasons leading to chlorosis of sugarcane plantlets.

Csatordayet al. reported inhibition of chlorophyll synthesis by Mn^2+^ in a cyanobacterium with accumulation of Mg protoporphyrin [34]. In tobacco callus, Mn^2+^ toxicity was also mediated by inhibition of chlorophyll synthesis [35]. Under excess Mn stress, wheat chlorophyll synthesis was also decreased [4]. In the present study, we found that the leaf chlorophyll concentration in newly emerged ratoo n plantlets in early spring was very low, leaves that were not fully expanded appeared yellow, and there was no obvious process from greening to yellowing (Fig 1). Instead, when the chlorotic plantlets entered the tillering stage, their chlorophyll concentration increased and the symptoms of chlorosis disappeared. These results suggest that the decreased chlorophyll concentration caused by excess Mn can be explained by inhibited chlorophyll synthesis.

The processes in the biosynthesis of chlorophyll that require Fe can be inhibited by Mn [35–38]. Mn^2+^-induced Fe deficiency is known to be a possible cause of reduced chlorophyll concentration in vascular plants [35, 36, 38]. Mn^2+^-induced Fe deficiency has been observed in soybean, pineapple, and red kidney beans [19, 39]. However, the incidence of Mn-induced Fe deficiency correlated poorly with Fe concentration in some species of plants such as soybean and pineapple [19, 40]. Various methods have been proposed to extract the fraction of total Fe that is metabolically active and is related to occurrence of Fe chlorosis [40, 41].In the present study, total Fe concentrations in chlorotic plantlets were at a normal level. However, active Fe from chlorotic plantlets was at a significantly lower level than that in the control plants (Fig 9A), which is consistent with the results of Lu, who reported that excess Mn led to reduced HCl extraction of physiologically active Fe in chlorotic pineapple leaves and that spraying with Fe fertilizer could alleviate leaf chlorosis [19].The lack of physiologically active Fe may result from the accumulation of physiologically inactive Fe pools in chlorotic leaves [19, 42, 43]. Thus, the lack of physiologically active Fe would be a secondary effect of Mn toxicity, which might block chlorophyll synthesis and thereby relate to chlorosis in sugarcane plantlets.

The degree of chlorosis, chlorophyll concentration and Mn concentration in sugarcane varied with growth periods and seasons (Fig 6). In the spring, sugarcanes are in the emergence and plantlet growth stages, the temperature and leaf chlorophyll concentration were low (S4 Fig and Fig 6), and the plantlets exhibited chlorosis. In early summer (May), the temperature rose rapidly, sugarcane plants developed into the late plantlet stage and early tillering stage, the leaf chlorophyll concentration greatly increased, and the symptoms of chlorosis in sugarcane plantlets disappeared. The phenomenon of harmful levels of Mn toxicity varying with changes in temperature was also found in other plants, such as soybean, tobacco, and oat. Low temperatures resulted in severe Mn toxicity to soybean [4, 44, 45]. Tobacco seedlings are more prone to Mn toxicity at lower temperatures [46]. Oat seedlings increased their resistance to Mn at higher temperatures [44]. Prevention of toxicity via warm temperature was associated with an increased rate of leaf expansion accompanied by increased vacuolar capacity for disposal of accumulated Mn^2+^ [4]. Mn concentration decrease in sugarcane accompanied by an increased rate of leaf expansion should be one of the explanations for the alleviation of chlorosis in the late growth stages at warm temperature.

Taken together, these findings suggest that chlorosis in ratoon sugarcane plantlets grown on acidic soils in South China is caused by excess Mn accumulated in parent stalks, while Mn-induced deficiency of active Fe may play a secondary role in the chlorosis.

## Supporting Information

S1 Fig Chlorotic plantlet and its neighbor plantlet without symptoms of chlorosis. The plantlets were grown in a field in Chongzuo, a city in southern China.
(TIF)

S2 Fig Sugarcane plantlets showing the difference in the incidence of chlorosis between planted cane sugarcane and ratoon sugarcane, grown in the same field in Chongzuo. (TIF)

S3 Fig Roots of chlorotic ratoon sugarcane plantlets grown in strongly acidic soil (pH 4.08) in Chongzuo. The figure depicts the inactive roots of the chlorotic plantlet.
(TIF)

S4 Fig Daily change of maximum and minimum atmospheric temperature between Jan 1 and Jul 31, 2014 in Chongzuo, Guangxi, China. (TIF)
